# Supplementary material for: The MiDAC histone deacetylase complex is essential for embryonic development and has a unique multivalent structure
Source: Nat Commun. 2020 Jun 26;11:3252. doi: 10.1038/s41467-020-17078-8 (PMC7319964; doi:10.1038/s41467-020-17078-8)
Supplement: Supplementary file 1 — Supplementary Information [file 41467_2020_17078_MOESM1_ESM.pdf]

The MiDAC histone deacetylase complex is essential for embryonic development and has a unique multivalent structure.

Robert E Turnbull[1,2]*, Louise Fairall[1,2]*, Almutasem Saleh[1,2,3]*, Emma Kelsall[2,4]*, Kyle L Morris[5,6], TJ Ragan[1], Christos G Savva[1], Aditya Chandru[2,7], Christopher J Millard[1,2], Olga V Makarova[2], Corinne J Smith[5], Alan M Roseman[8], Andrew M Fry[2], Shaun M Cowley[2]+ and John WR Schwabe[1,2]+

* These authors contributed equally

+ Co-corresponding authors: smc57@leicester.ac.uk; john.schwabe@leicester.ac.uk

1. Leicester Institute of Structural and Chemical Biology, University of Leicester, LE1 7RH, UK
2. Department of Molecular and Cell Biology, University of Leicester, LE1 7RH, UK
3. Present address: Institute of Clinical Sciences, Faculty of Medicine, Imperial College London, Hammersmith Hospital Campus, Du Cane Road, London, W12 0HS, UK
4. Present address: AstraZeneca, Milstein Building, Granta Park, Cambridge, CB21 6GH, UK
5. School of Life Sciences, University of Warwick, Coventry, CV4 7AL, UK
6. Present address: MRC London Institute of Medical Sciences, Hammersmith Hospital Campus, Du Cane Road, London, W12 0NN, UK
7. Present address: Department of Genetics, University of Cambridge, Downing Street, Cambridge CB2 3EH, UK
8. Division of Molecular and Cellular Function, University of Manchester, M13 9PL, UK.

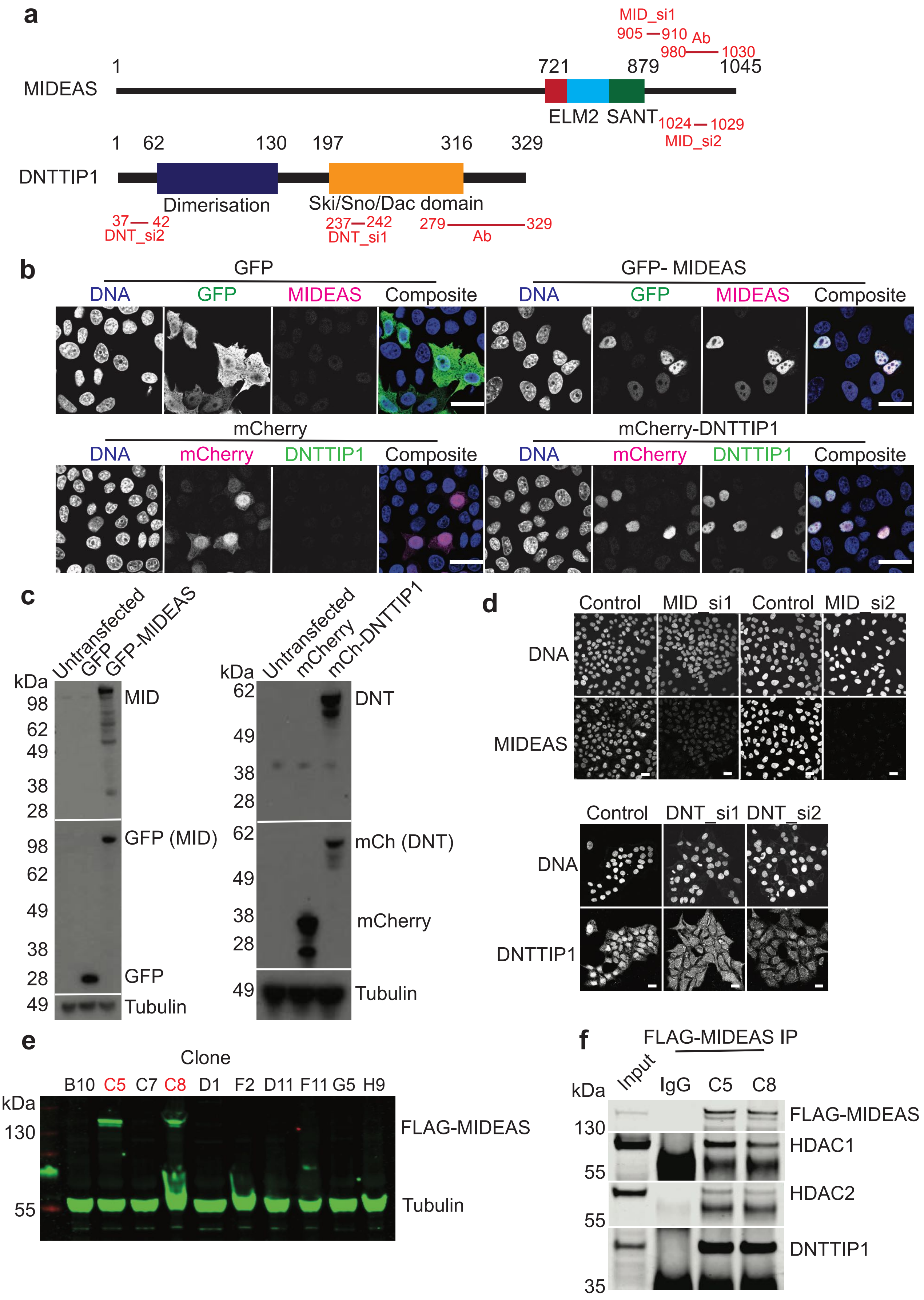
a
MID_si1
905 910
Ab
980 1030
1 721 879 1045
MIDEAS
ELM2 SANT
1024 1029
MID_si2
1 62 130 197 316 329
DNTTIP1
Dimerisation
Ski/Sno/Dac domain
37 42
DNT_si2
237 242
DNT_si1
279 329
Ab
b
GFP
GFP- MIDEAS
DNA GFP MIDEAS Composite
mCherry
mCherry-DNTTIP1
DNA mCherry DNTTIP1 Composite
c
Untransfected
GFP
GFP-MIDEAS
kDa
98 62 49 38 28
MID
GFP (MID)
GFP
Tubulin
Untransfected
mCherry
mCh-DNTTIP1
62 49 38 28
DNT
mCh (DNT)
mCherry
Tubulin
d
Control MID_si1 Control MID_si2
DNA
MIDEAS
Control DNT_si1 DNT_si2
DNTTIP1
e
Clone
B10 C5 C7 C8 D1 F2 D11 F11 G5 H9
kDa
130
55
FLAG-MIDEAS
Tubulin
f
FLAG-MIDEAS IP
Input IgG C5 C8
130 55 55 35
FLAG-MIDEAS
HDAC1
HDAC2
DNTTIP1

Supplementary Figure 1. Antibodies and siRNA against MIDEAS and DNTTIP1 are specific.
a Schematic of MIDEAS and DNTTIP1 protein domain organisation. The antigenic region for antibody binding and target sequence for siRNA are shown.
b Confocal images of U2OS cells transfected with plasmids coding either GFP, GFP-MIDEAS, mCherry or mCherry-DNTTIP1 and dual stained with either GFP (green)/MIDEAS (magenta) or mCherry (magenta)/DNTTIP1 (green). DNA (blue) was visualised with Hoechst 33258 (Scale: 25 μm). (Representative image of n=2 independent experiments)
c Western blot for GFP, MIDEAS (MID), mCherry, DNTTIP1 (DNT) and Tubulin from lysates prepared from U2OS cells transfected with plasmids as in panel b. (n=1)
d Confocal images of PFA fixed U2OS cells following treatment with siRNA for 72 h targeting MIDEAS (top) and DNTTIP1 (bottom). Cells were stained for either MIDEAS or DNTTIP1. DNA was visualised with Hoechst 33258 (scale: 25 μm). (Representative image. Nuclear staining intensity was measured using FIJI of a minimum of 300 cells across 3 independent experiments)
e Western blot for FLAG epitope from cells that had screened postive for successful CRISPR of the FLAG epitope at the C-terminus of the MIDEAS gene in mouse ES cells. Clones in red show FLAG epitope at correct MW for MIDEAS-FLAG protein. (n=1)
f Western blot for HDAC1, HDAC2, DNTTIP1 and FLAG following Co-IP using a FLAG antibody from CRISPR MIDEAS-FLAG mES cell lysates. (n=1) Source data are provided in the Source data file.

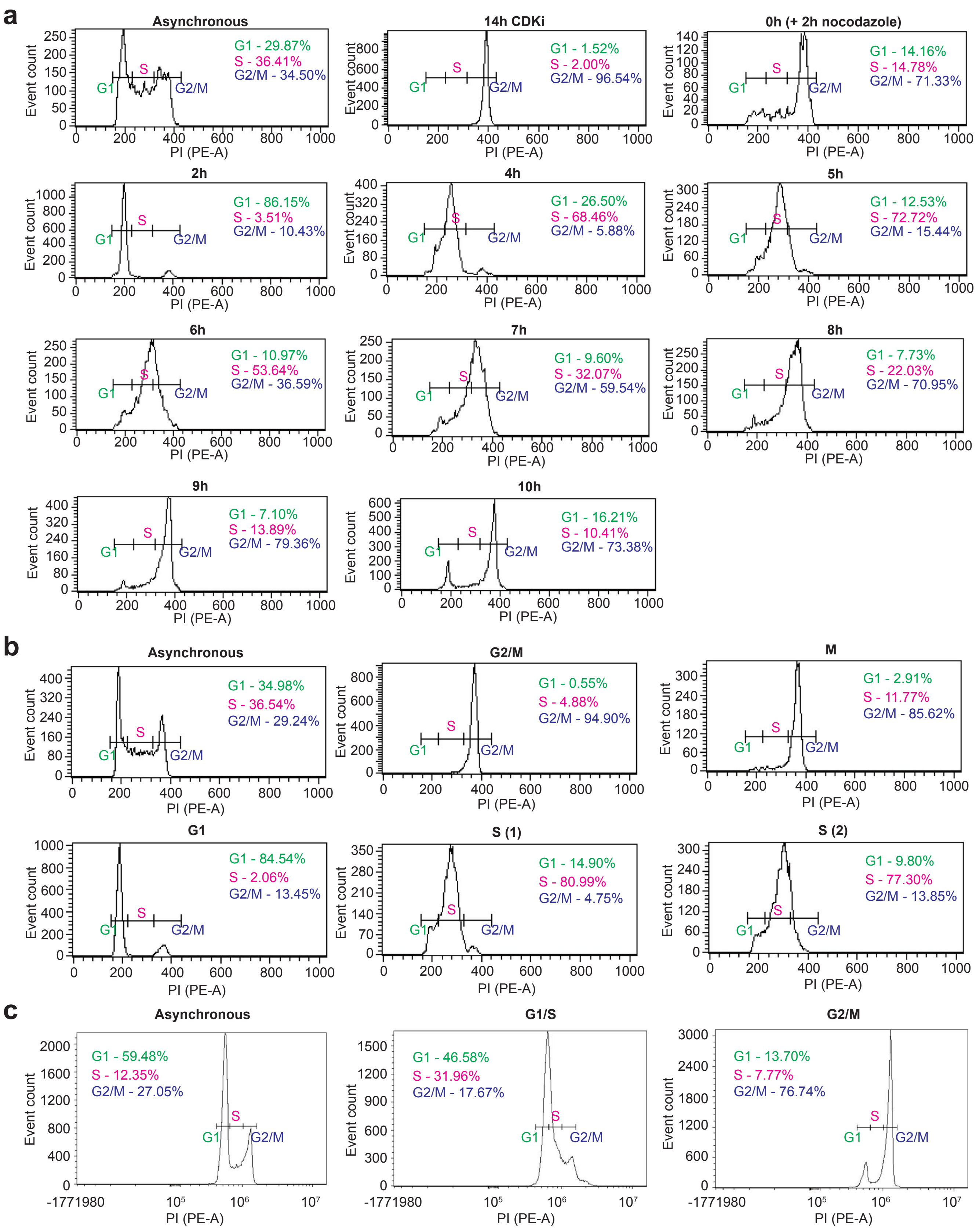

Supplementary Figure 2. Representative flow cytometry graphs of synchronised cells.
a Flow cytometry plots for Fig.1**e**. Cells were blocked for 14 h with the Cdki RO3306 (10 μM) and then further blocked with nocodazole (30 ng/ml) for 2 h. After release from the nocodazole block, cells were harvested at the indicated time points to capture progression through the cell cycle.
b Flow cytometry plots for Fig. 1**f**. Cells were synchronised as above and after release cells were harvested at the indicated cell cycle phase and lysates used for FLAG IP.
c Flow cytometry plots for Fig. 1**g**-**h**. Asynchronous or cells blocked with aphidicolin (1.6 μg/ml) (G1/S) or nocodazole (3.3 μg/ml) (M) for 16 h were harvested and nuclear lysates used for MIDEAS or DNTTIP1 IP's followed by an HDAC activity assay.
All data were analysed and plots created using FCSalyzer v0.9.15.

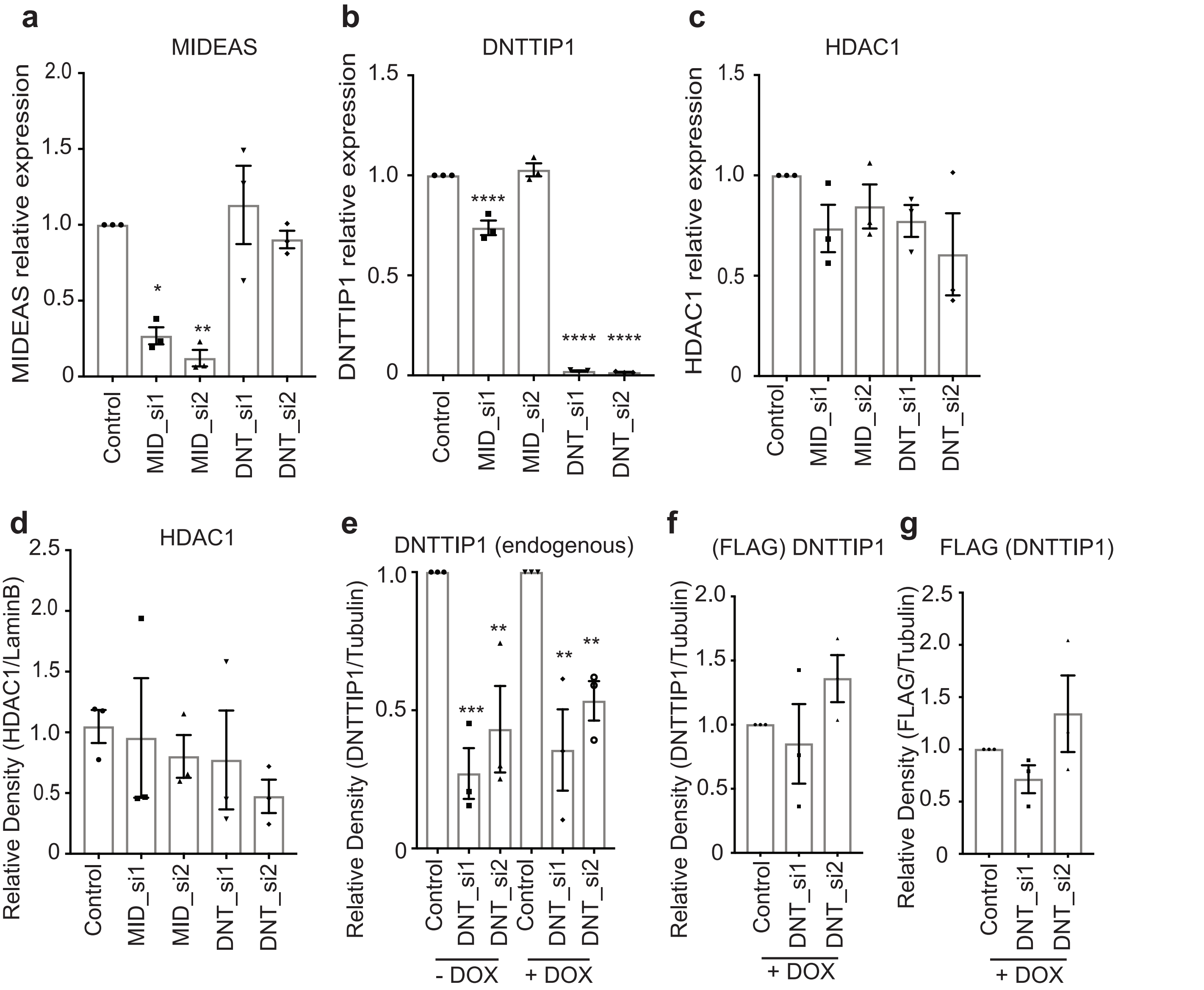

Supplementary Figure 3. Effect of siRNA on MiDAC mRNA and protein expression and FLAG-DNTTIP1 rescue.
a-c qPCR analysis of MIDEAS (**a**), DNTTIP1 (**b**) and HDAC1 (**c**) following treatment of U2OS cells with siRNA against MIDEAS or DNTTIP1 for 72 h. Relative expression was calculated using the ΔΔCT method relative to the housekeeping gene B2-microglobulin and normalised to the control samples (Mean ± s.e.m, n=3 independent experiments, *p<0.01 (**a** P=0.0061 for control versus MID_si1, 0.0017 for control versus MID_si2), ****p<0.0001, one-way ANOVA with Dunnett's post hoc test).
d Quantification of HDAC1 protein levels in U2OS cells following treatment with siRNA for 72 h targeting MIDEAS or DNTTIP1. LaminB was detected in parallel from the same lysate sample and was used as a reference for normalisation (Mean ± s.e.m, n=3 independent experiments).
e-g Quantification of DNTTIP1 (**e**) and FLAG-DNTTIP1 (**f**,**g**) protein levels in U2OS cells following treatment with siRNA for 72 h targetting DNTTIP1. After 48 h, expression of the siRNA resistant FLAG-DNTTIP1 was induced by DOX. Alpha-tubulin was detected in parallel from the same lysate sample and was used as a reference for normalisation (Mean ± s.e.m, n=3 independent experiments, **p<0.01 (**a** p=0.0033 for control versus DNT_si2 w/o DOX, 0.002 for control versus DNT_si1 with DOX, 0.0063 for control versus DNT_si2 with DOX), ***p=0.0009, one-way ANOVA with Holm-Sidak post hoc test).
Source data are provided in the Source data file.

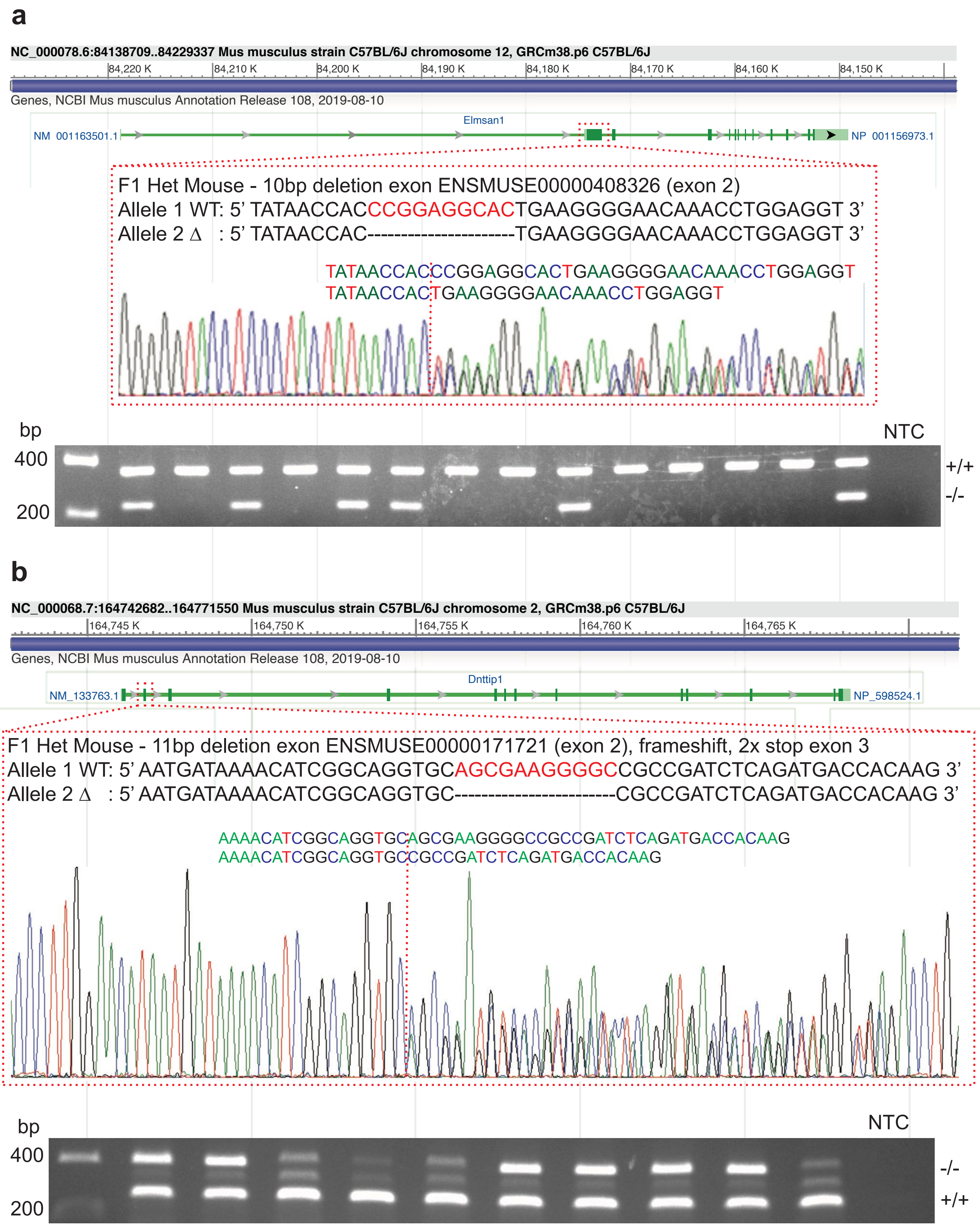

Supplementary Figure 4. CRISPR-targetted deletions in MIDEAS and DNTTIP1 and representative genotyping.

a Organisation of MIDEAS exon and intragenic regions. CRISPR-targetted sequence is in exon 2 (ENSMUSE00000408326). Sequencing data is from first generation heterozygous mice. Agarose gel shows representative genotyping of litters from Het x Het mates with the wildtype (top) and deletion (bottom) alleles clearly distinguishable.

b Organisation of DNTTIP1 exon and intragenic regions. CRISPR-targetted sequence is in exon 2 (ENSMUSE00000171721). Sequencing data is from first generation heterozygous mice. Agarose gel shows representative genotyping of litters from Het x Het mates with the wildtype (bottom) and deletion (top) alleles clearly distinguishable.

Source data are provided in the Source data file.

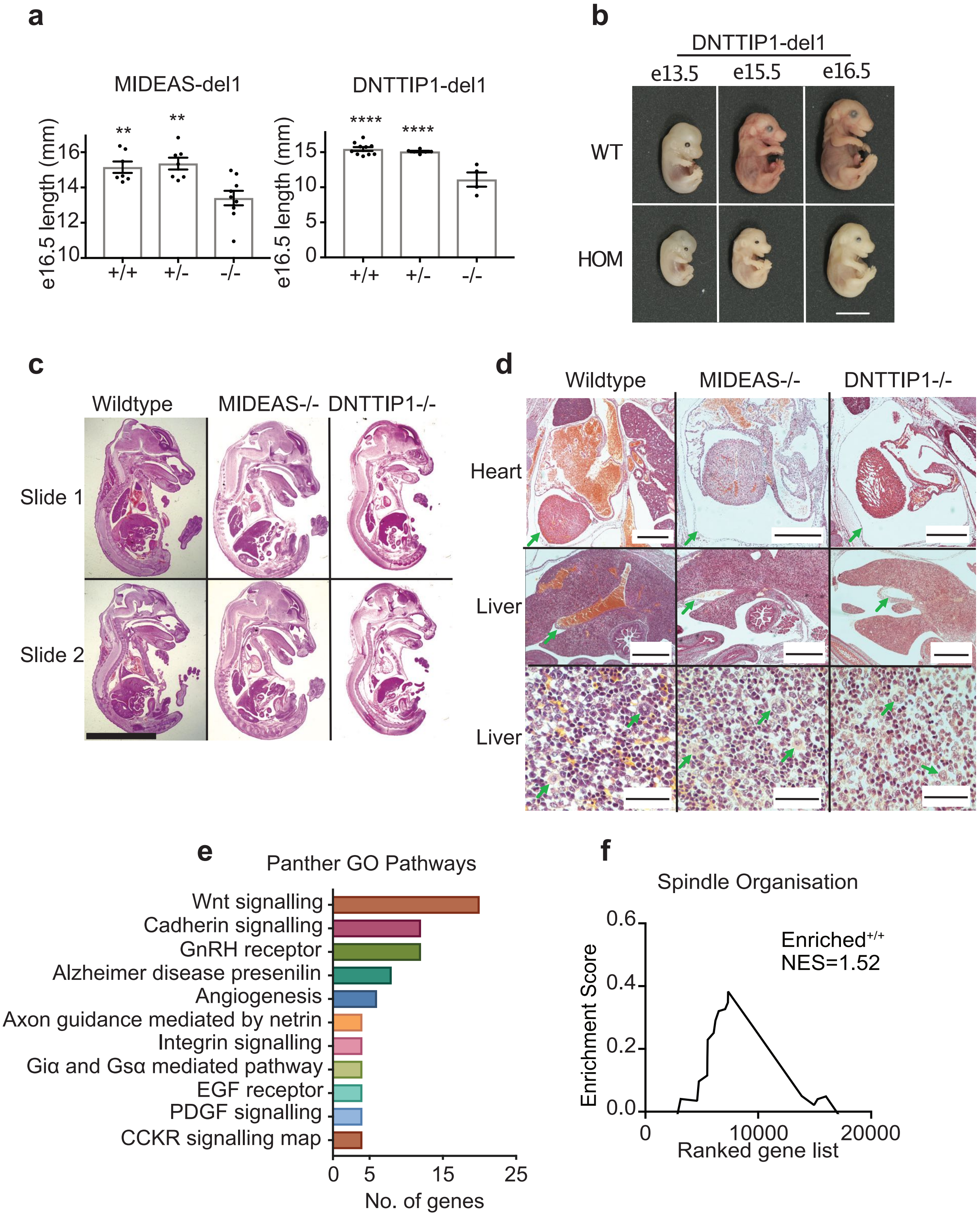

Supplementary Figure 5. Histology of mouse embryo heart and liver and MEF RNAseq pathway and GSEA.
a Quantification of embryo length from e16.5 embryos. Embryo size was calculated using FIJI by drawing a line from the top of the embryo head to its bottom. (Mean ± s.e.m, n=7, 7, 9 (MIDEAS-del1 +/+, +/-, -/- respectively), n=10, 6, 4 (DNT-TIP1-del1 +/+, +/-, -/- respectively), **p<0.01 (p=0.008 for +/+ versus -/-, 0.0033 for +/- versus -/-), ****p<0.0001, one- way ANOVA with Tukey's post hoc test).
b Comparison of DNTTIP1-del1 wildtype and homozygous embryo's at e13.5, e15.5 and e16.5. Scale bar = 5 mm.
c Images of whole embryos stained for H&E. Overall the knockout embryos look normal in terms of limb development compared to wildtype. (Representative images from n=2 biologically independent animals). Scale bar = 5 mm.
d Images of sections from wildtype, MIDEAS-/- and DNTTIP1-/- e16.5 embryos. Images show lack of erythrocytes in the heart and rounded shape with an enlarged pericardium and deformed ventricle morphology in the knockout compared to wildtype (arrows). Overall morphology of the liver and close up showing similar distribution and presence of haematopoietic cells in both. (Representative images from n=2 biologically independent animals). Scale bar upper six panels = 500 µm. Scale bar lower three panels = 50 µm.
e Functional classification of the overlapping, upregulated gene set using PANTHER (Protein Analysis Through Evolution-ary Relationships) (http://www.pantherdb.org).
f GSEA comparing MIDEAS and DNTTIP1 knockout MEFs to wildtype. (NES; normalised enrichment score, calculated by the GSEA software using default parameters (www.broad.mit.edu/gsea)).
Source data provided in the Source data file.

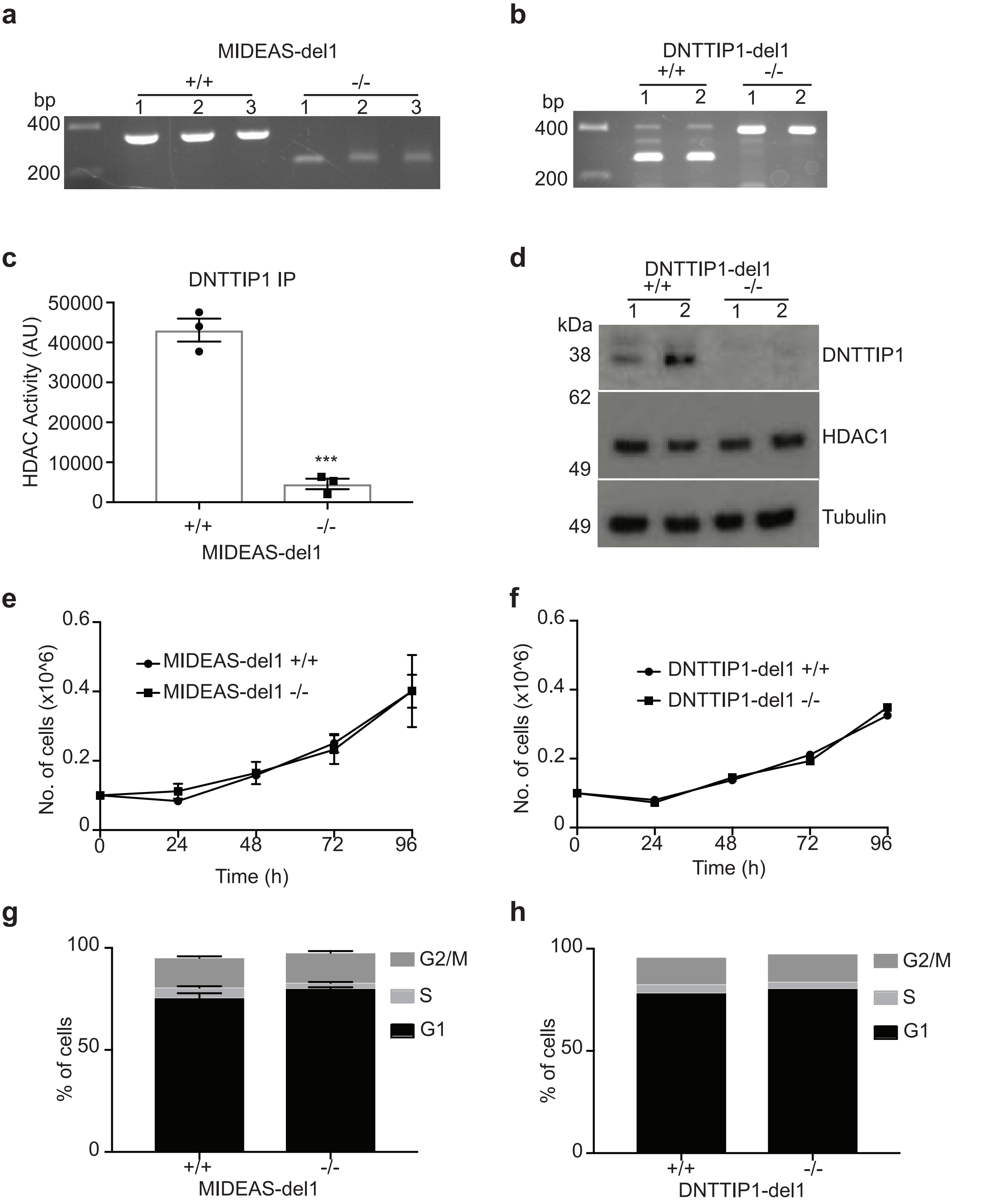

Supplementary Figure 6. Characterisation of MIDEAS-del1 and DNTTIP1-del1 MEF lines.
a,b Genotyping results for wildtype and homozygous e13.5 MEFs from MIDEAS-del1 (**a**) and DNTTIP1-del1 (**b**) embryos. (n=3 **a**, n=2 **b**, from biologically independent cells isolated from e13.5 embryos)
c Fluorescent HDAC activity assay from DNTTIP1 IP using nuclear protein lysates from wildtype and homozygous knockout MIDEAS-del1 MEFs. (Mean ± s.e.m, n=3 biologically independent cells isolated from e13.5 embryos, ***p=0.0003, two-tailed unpaired Student's t-test).
d Western blot for DNTTIP1, HDAC1 and tubulin using nuclear protein lysates from wildtype and homozygous knockout DNTTIP1-del1 MEFs. (n=2 from biologically independent cells isolated from e13.5 embryos).
e,f Proliferation over 96 h for wildtype and homozygous MEFs from MIDEAS-del1 (**e**) and DNTTIP1-del1 (**f**) lines. Cells ($0.1x10^6$) were seeded into wells and every 24 h detached using trypsin and counted using a BioRad automated cell counter. (Mean ± s.e.m, n=3 **e**, n=2 **f**, from biologically independent cells isolated from e13.5 embryos).
g,h Cell cycle distribution of wildtype and homozygous knockout MIDEAS-del1 (**g**) and DNTTIP1-del1 (**h**) MEFs. (Mean ± s.e.m, n=3 (MIDEAS-del1), n=2 (DNTTIP1-del1) from biologically independent cells isolated from e13.5 embryos).
Source date provided in the Source data file.

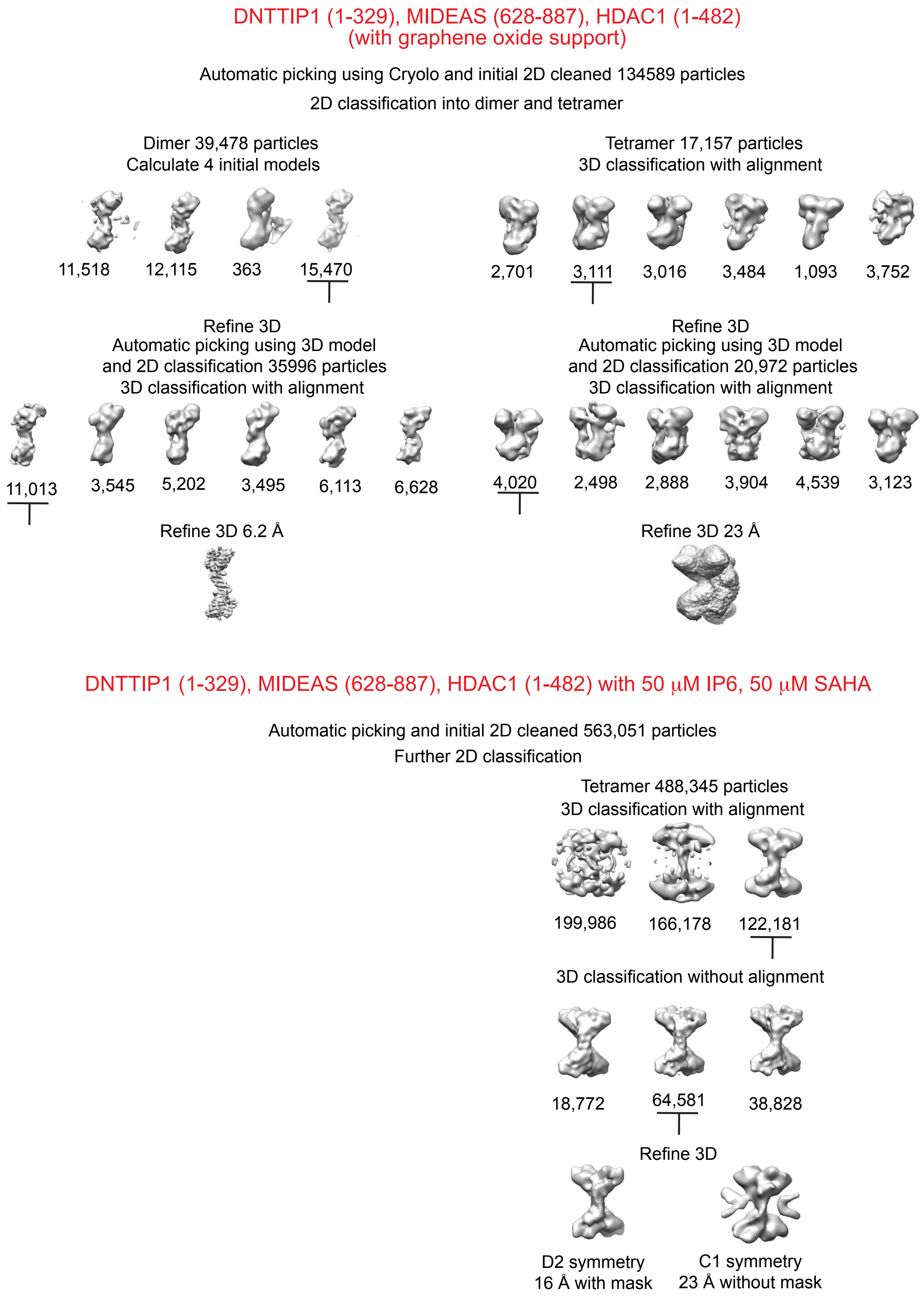

Supplementary Figure 7. Summary of the image processing procedures followed in Relion3 for the dimer and tetramer including the DNTTIP1 DNA-binding domain

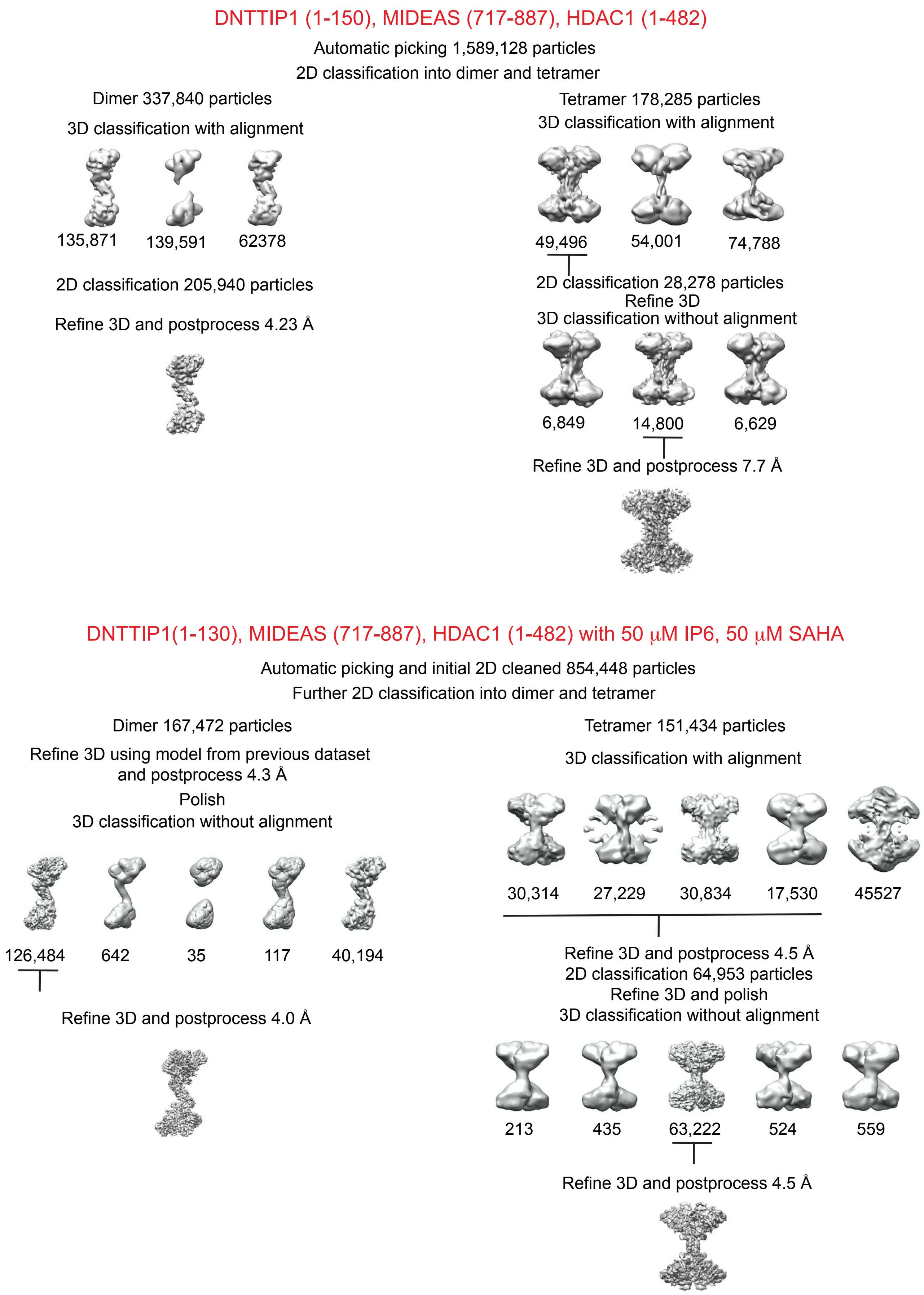

Supplementary Figure 8. Summary of the image processing procedures followed in Relion3 for the dimer and tetramer lacking the DNTTIP1 DNA-binding domain

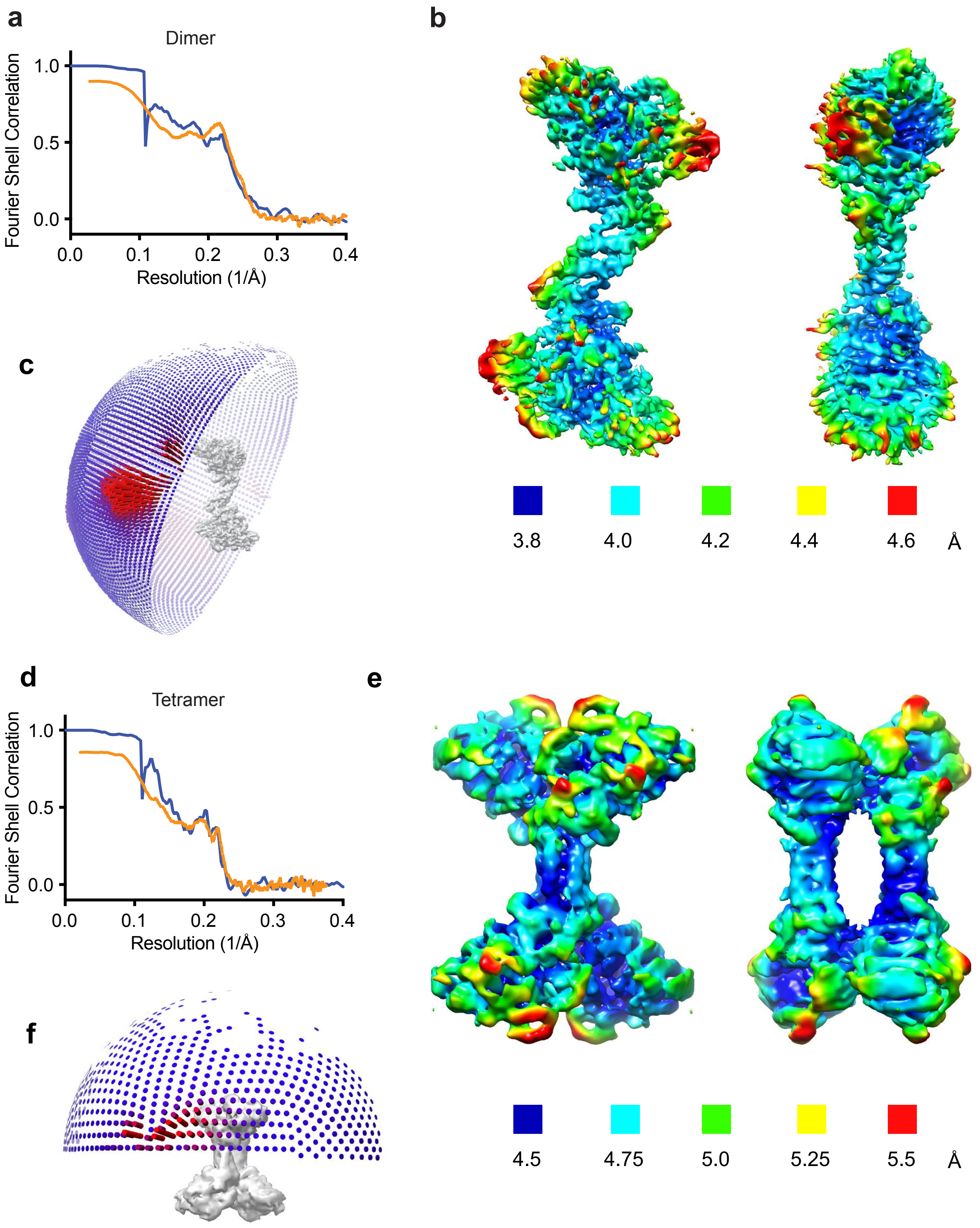

Supplementary Figure 9. Resolution assessment for the MiDAC complexes
a Fourier Shell Correlation between the two half-maps (blue) and between the Postprocess map from Relion and the refined model (orange) for the dimer.
b Electron density map of the Refine3D map from Relion3 coloured by resolution for the dimer.
c Angular distribution of particles for the Refine3D map of the dimer using C2 symmetry.
d Fourier Shell Correlation between the two half-maps (blue) and between the Postprocess map from Relion and the refined model (orange) for the tetramer.
e Electron density map of the Refine3D map from Relion3 coloured by resolution for the tetramer.
f Angular distribution of particles for the Refine3D map of the tetramer using D2 symmetry.
Source data are provided in the Source data file.

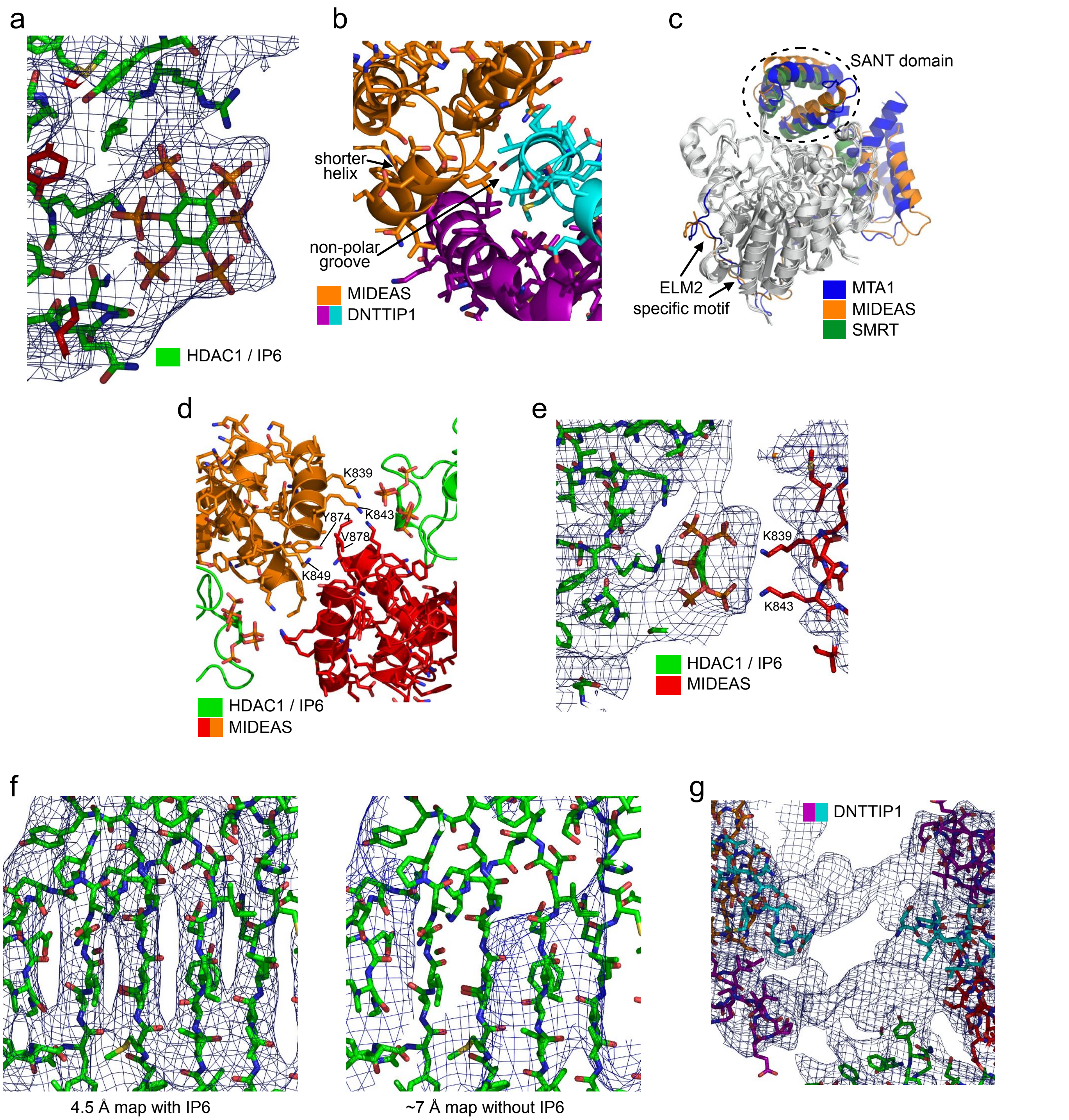

Supplementary Figure 10. Details of the MiDAC structure, maps and comparisons

a Close up of the 4.5 Å map of the tetrameric complex showing IP6.

b View of the interaction between MIDEAS and the non-polar groove in the DNTTIP1 dimerisation domain.

c Comparison of the SANT domain in MIDEAS with MTA1:HDAC1 (PDB: 5icn) and SMRT:HDAC3 (PDB: 4a69). The ELM2 specific motif common to MIDEAS and MTA1 (but not SMRT) is highlighted.

d Close up of the interactions between the MIDEAS SANT domains in the tetramer.

e Lysine residues that are likely to mediate additonal tetrameric interactions with IP6 are highlighted. Note that density for these was not seen in the 4.5 Å map.

f Comparison of the maps around the core of the HDAC1 enzyme in tetrameric complex with and without IP6.

g Evidence in the 4.5 Å map that the amino-terminal region of DNTTIP1 crosses over, contributing to tetrameric interactions.

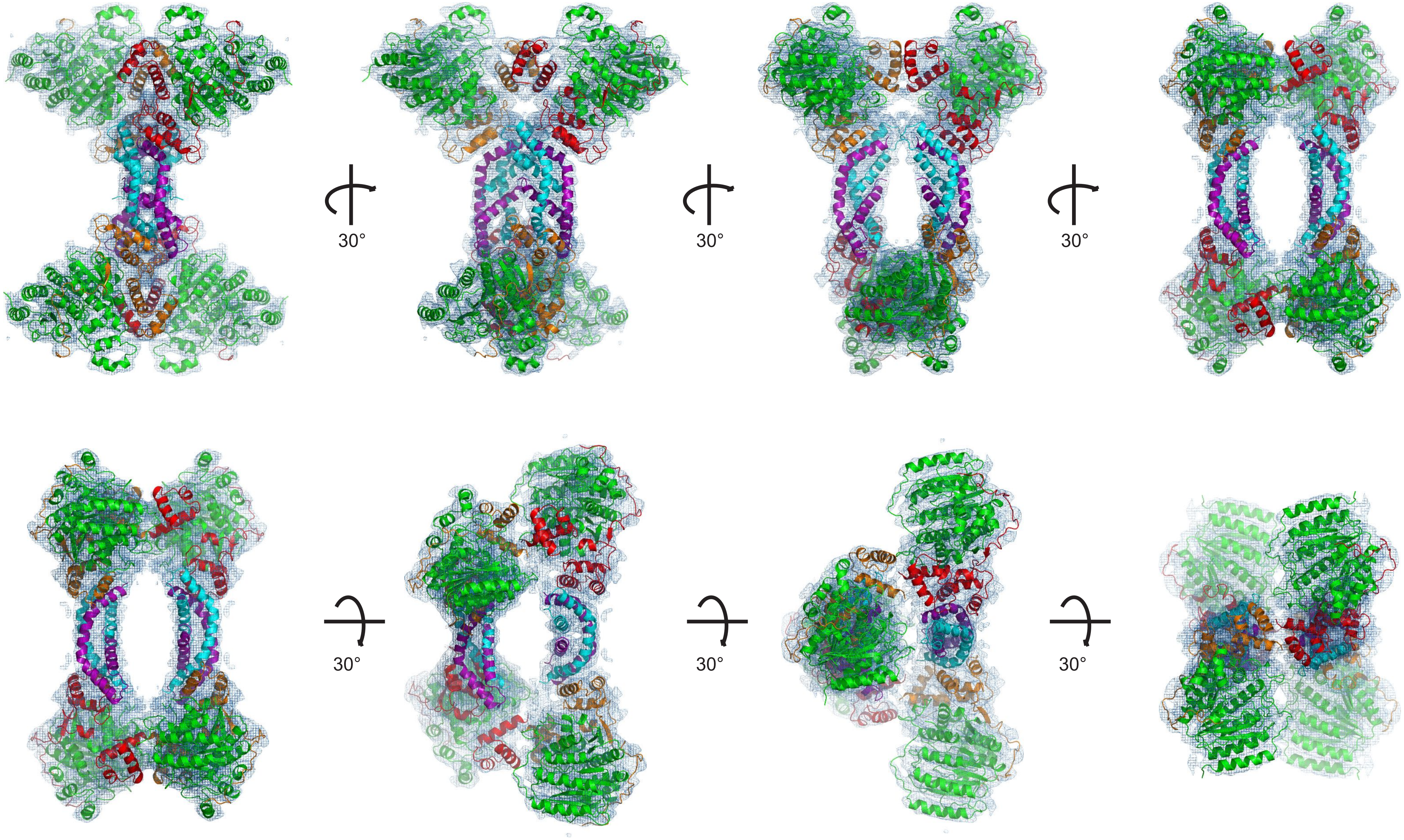

Supplementary Figure 11. Postprocess map from Relion3 of tetramer.

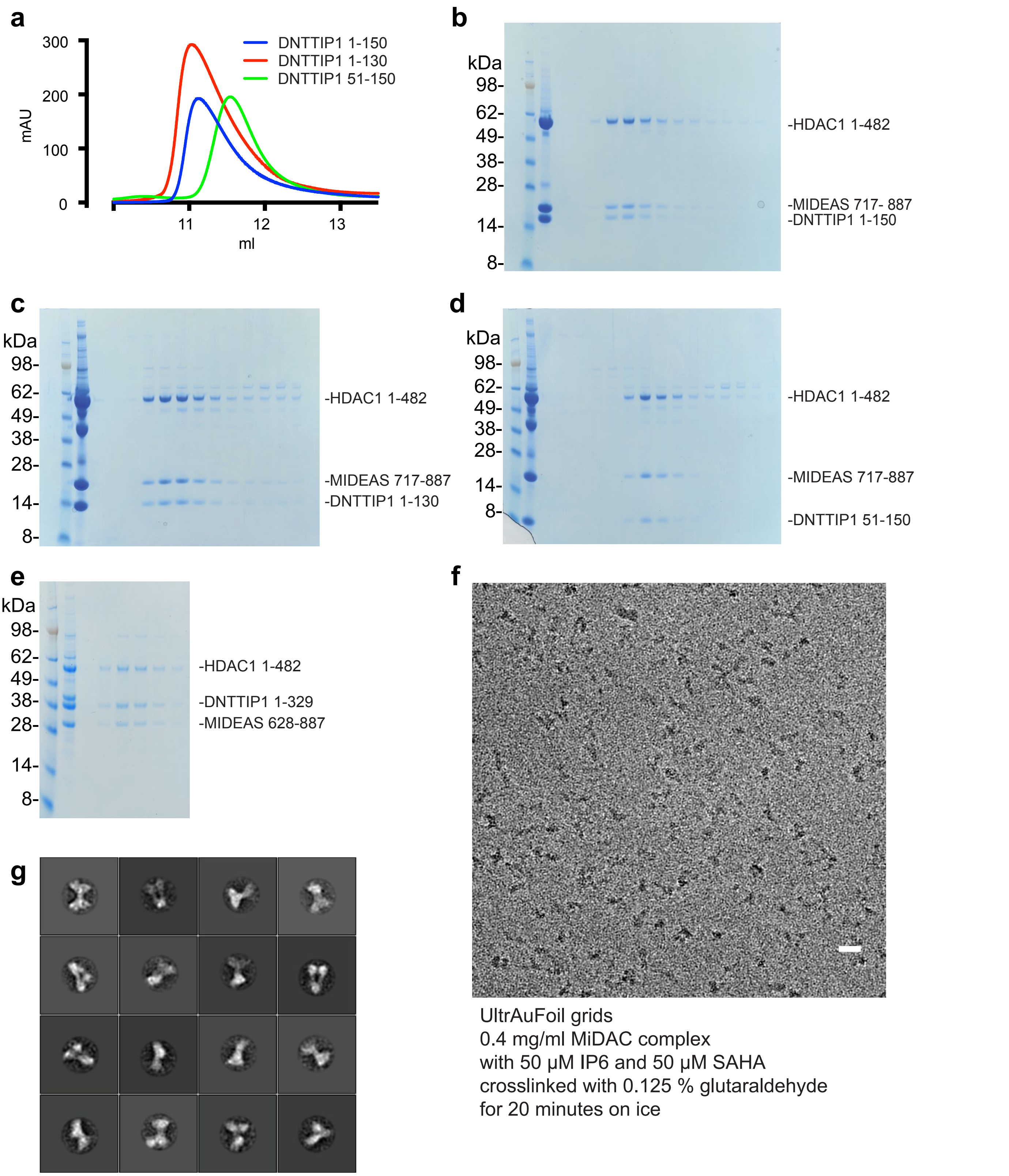

Supplementary Figure 12. Analysis of MiDAC complexes with different length constructs of DNTTIP1
a Gel filtration profiles of the complex from a Superdex S-200 column with DNTTIP1 (1-150), (1-130) and (51-150)
b-d SDS-PAGE of the complexes from a Superdex S-200 column with DNTTIP1 (1-150), (1-130) and (51-150)
e SDS PAGE of the gel filtration purification from a Superdex S-200 gel filtration column of the complex with full-length DNTTIP1 containing the DNA-binding domain.
f Electron micrograph of the complex with full-length DNTTIP1 containing the DNA-binding domain. Scale bar = 20nm.
g 2D class averages of the complex with full-length DNTTIP1 containing the DNA-binding domain.
Source data are provided in the Source data file.

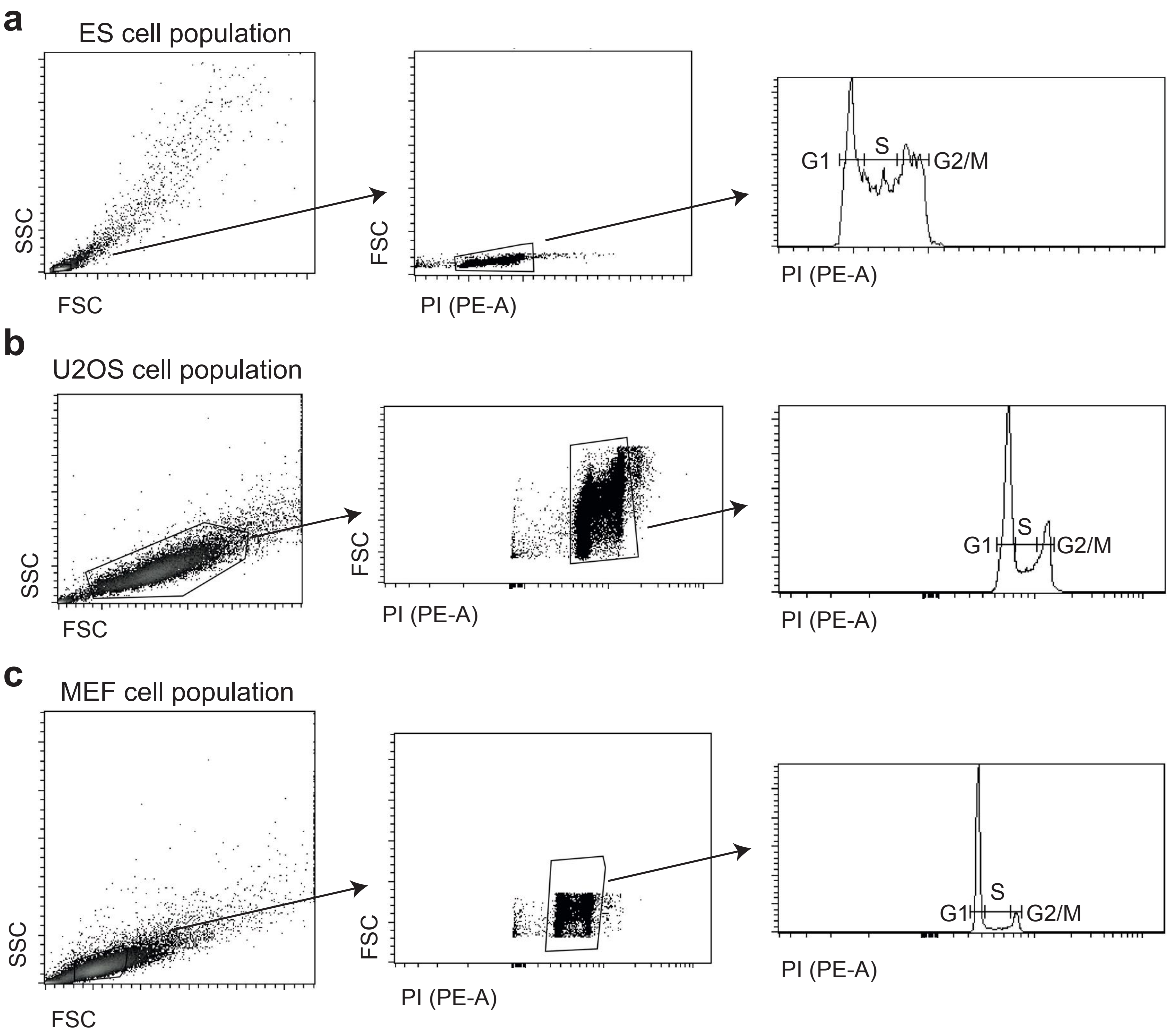

Supplementary Figure 13. Gating strategies used for flow cytomtery.
a Gating strategy to determine cell cycle distribution of ES cells presented in Fig. 1**e**,**f**.
b Gating strategy to determine cell cycle distribution of U2OS cells presented in Fig. 1**g**,**h**.
c Gating strategy to determine cell cycle distribution of MEF cells presented in Supp. Fig. 6**g**,**h**.

Line	Wildtype	Heterozygous	Homozygous	Total	Male:Female	Male:Female (Het)	No. of Litters	Avg. Litter Size
**MIDEAS-del1**	44	64	0	108	48:58 (NS)	32:32 (NS)	18	6
**e13.5**	7	18	7	32				
**e15.5**	2	11	4	17				
**e16.5**	8	11	10	29				
**DNTTIP1-del1**	24	52	0	76	31:45 (NS)	20:32 (NS)	15	5.1
**e13.5**	4	16	3	23				
**e15.5**	6	12	5	23				
**e16.5**	12	22	6	40				
**e17.5**	4	6	0	10				

Supplementary Table 1. Genotyping of mouse embryos and litters from MIDEAS +/- or DNTTIP1 +/- mates

DAVID				Panther			
**Wnt signalling**	**Adhesion**	**Axon Guidance**	**Angiogenesis**	**Wnt / Cadherin signalling**	**Axon guidance**	**EGF/FGF**	**Angiogenesis**
Fzd4	Atplb2	Gli2	Angpt2	Fzd4	Pik3r1	Shc3	Pik3r1
Fzd9	Ephb4	Apbb1	Tek	Fzd9	Dcc	Mras	Angpt2
Frzb	Fat3	Ank3	Vash2	Frzb	Ntn1	Map2k6	Sfrp1
Wnt11	Frem1	Boc		Wnt11	Ntn3	Map3k5	Frzb
Wnt2	Adam23	Bmp7		Wnt2			Wnt2
Amer1	Ackr3	Cdh4		Celsr1			Tek
Dixdc1	Boc	Dcc		Pcdh10			
CDK14	Cdh4	Dix5		Pcdh18			
Lgr4	Cdh6	Efna3		Pcdhb19			
Lrp4	Cdh8	Efna5		Plcb1			
Nkd1	Cdh26	Ntn1		Pygo1			
Pygo1	Celsr1	Ntn3		Arrb1			
Ror1	Col6a6	Sema5a		Smad9			
Sfrpl1	Cntn1			Dchs1			
Sfrpl4	Dchs1			Cdh4			
Tcf7l1	Emilin2			Cdh6			
	Fbtn7			Cdh8			
	Kit1			Sfrp1			
	Nid2			Sfrp4			
	PlcBl2						
	Pcdh10						
	Pcdh18						
	Span2						

Supplementary Table 2. Upregulated genes identified to play roles in development

MIDEAS		DNTTIP1		Overlapping	
**Down**	**Up**	**Down**	**Up**	**Down**	**Up**
Tubulin alpha 4a	Septin 3	Tubulin alpha 4a	Septin 3	Tubulin alpha 4a	Septin 3
cDNA sequence BC034090	Septin 4	cDNA sequence BC034090	Septin 4	cDNA sequence BC034090	Septin 4
	Septin 6		Septin 6		Septin 6
Pard6b	CDKi 1C		CDKi 1C		CDKi 1C
Actin, alpha 1	Ubiquitin specific peptidase 29		Ubiquitin specific peptidase 29		Ubiquitin specific peptidase 29
Actin, gamma-enteric					
	MAP, RP/EB fmily, member 2		MAP, RP/EB fmily, member 2		MAP, RP/EB fmily, member 2
Polo-like kinase 2					
Myosin VC	Tubulin delta 1		Timeless		
Myosin 1C	MT associated protein (MAP) 10		Cell Division Cycle 6		
Tubulin alpha 4a			Wee1		
Tubulin alpha 8	IL1R associated kinase 3		Anillin		
Tubulin beta 6			Rhotekin 2		
IL1R associated kinase 1			Cyclin E2		
			Rad21		
DNA excision repair protein ERCC1			Tubulin epsilon 1		
			Cyclin D1		
			SPC25		
			F-box only protein 5		
			DNA Topoisomease II alpha		
			non-SMC condensin 1 complex		

Supplementary Table 3. Cell cycle associated genes up and down regulated in knockouts

Gene Symbol	Gene title	Rank in gene list	Rank metric score	Running ES	Core enrichment
CHMP4C	Chromatin modifying protein 4C	296	0.93	0.19	Yes
PARP3	Poly (ADP-ribose) polymerase family member 3	1855	0.43	0.20	Yes
CHMP3	Chromatin modifying protein 3	2224	0.38	0.27	Yes
CHMP18	Chromatin modifying protein 18	3471	0.28	0.26	Yes
PSRC1	Proline/serine-rich coiled-coil 1	3611	0.27	0.32	Yes
RNF4	Ring finger protein 4	4235	0.22	0.33	Yes
CHMP5	Chromatin modifying protein 5	4248	0.22	0.38	Yes
CHMP1A	Chromatin modifying protein 1A	4274	0.22	0.43	Yes
TPR	Translocated promotoer region	4723	0.19	0.45	Yes
PDCD6IP	Programmed cell death 6 interacting protein	4865	0.18	0.48	Yes
CHMP2A	Chromatin modifying protein 2A	5102	0.17	0.51	Yes
SENP6	SUMO1/sentrin specific peptidase 6	5592	0.14	0.51	Yes
BORA	null	5810	0.13	0.53	Yes
CHMP4B	Chromatin modifying protein 4B	5815	0.13	0.56	Yes
VPS4B	Vacuolar protein sorting 4 homolog B	7741	0.035	0.46	No
PKD1	Polycystic kidney disease 1	11397	-0.06	0.28	No
TPX2	TPX2, microtubule-associated, homolog	12327	-0.12	0.25	No
PLK1	Polo-like kinase 1	12592	-0.14	0.27	No
CHMP2B	Chromatin modifying protein 2B	13226	-0.19	0.28	No

Supplementary Table 4. GSEA for spindle organisation showing enrichment in wildtype samples

	Dimer<br>(EMD-11041)<br>(PDB 6Z2J)	Tetramer<br>(EMD-11042)<br>(PDB 6Z2K)
**Data collection and processing**		
Magnification	75,000	75,000
Voltage (kV)	300	300
Electron exposure (e–/Å^2)	35.0	35.0
Defocus range (μm)	-0.5	-0.5
Pixel size (Å)	1.08	1.08
Symmetry imposed	C2	D2
Initial particle images (no.)	337,840	178,285
Final particle images (no.)	126,484	63,222
Map resolution (Å)	4.0	4.5
FSC threshold	0.143	0.143
Map resolution range (Å)	4.0-5.8	4.5-5.5
**Refinement**		
Initial model used (PDB code)	5ICN, 4D6K	5ICN, 4D6K
Model resolution (Å)	3.90	4.37
FSC threshold	0.143	0.143
Model resolution range (Å)		
Map sharpening *B* factor (Å^2)	-70	-127
Model composition		
Non-hydrogen atoms	9,613	19,298
Protein residues	1,186	2,373
Ligands	8	16
B factors (Å^2)		
Protein	81.4	239.6
Ligand	91.4	234.1
R.m.s. deviations		
Bond lengths (Å)	0.003	0.004
Bond angles (°)	0.743	0.824
Validation		
MolProbity score	1.44	1.57
Clashscore	1.91	2.56
Poor rotamers (%)	0.2	0.15
Ramachandran plot		
Favored (%)	92.12	90.84
Allowed (%)	7.88	8.95
Disallowed (%)	0	0.21

Supplementary Table 5: Cryo-EM data collection, refinement and validation statistics

Construct	
Untagged<br>DNTTIP1 1-329	Forward<br>ACCCAAGCTTGGTACCATGGGAGCCACTGGCGACGCC TDIF1_LF_4<br>Reverse<br>GACGGAGCTCGAATTTCAGGTCTGTGGAGGTGCTTCAAC TDIF1_LF_3
Untagged<br>DNTTIP1 1-150	Forward<br>ACCCAAGCTTGGTACCATGGGAGCCACTGGCGACGCC TDIF1_LF_4<br>Reverse<br>GACGGAGCTCGAATTTCAACGGCCACGCTTTATTCCTGG TDIF1_LF_7
Untagged<br>DNTTIP1 51-150	Forward<br>ACCCAAGCTTGGTACCATGGGCCGCCGCTCACAGATGACA TDIF1_LF_14<br>Reverse<br>GACGGAGCTCGAATTTCAACGGCCACGCTTTATTCCTGG TDIF1_LF_7
Untagged<br>DNTTIP1 1-130	Forward<br>ACCCAAGCTTGGTACCATGGGAGCCACTGGCGACGCC TDIF1_LF_4<br>Reverse<br>GACGGAGCTCGAATTTCATGAAAAGAGCAGTTTAGCCTG TDIF1_LF_15
Untagged<br>HDAC1 1-482	Forward<br>ACCCAAGCTTGGTACCATGGCGCAGACGCAGGGCACC HDAC1_CM_4<br>Reverse<br>GACGGAGCTCGAATTTTAGGCCAACTTGACCTCCTCCTT HDAC1_CM_3
Flag tagged<br>MIDEAS 628-887	Forward<br>GTATTTTCAGGGCGCCACCCCATACCAGAGCCACCTG MIDEAS_LF_13<br>Reverse<br>GACGGAGCTCGAATTTTAGGTTAGAGTCCCATTGCGGCC MIDEAS_LF_9
Flag tagged<br>MIDEAS 717-887	Forward<br>GTATTTTCAGGGCGCCGTGAGCATCGAGCCACGGATC MIDEAS_LF_15<br>Reverse<br>GACGGAGCTCGAATTTTAGGTTAGAGTCCCATTGCGGCC MIDEAS_LF_9
GFP tagged<br>MIDEAS 1-1019	Forward<br>TCCGGACTCAGATCTATGAACCTCCAGGCCCAGCCC MIDEAS_RT_1<br>Reverse<br>GTCGACTGCAGAATTTTATGAAAAAGGTAGTGCCCTTCG MIDEAS_RT_2
mCherry tagged<br>DNTTIP1 1-329	Forward<br>TCCGGACTCAGATCTATGGGAGCCACTGGCGACGCC TDIF1_RT_1<br>Reverse<br>GTCGACTGCAGAATTTCAGGTCTGTGGAGGTGCTTC TDIF_RT_2
PiggyBac<br>FLAG tagged<br>DNTTIP1 1-329	Forward<br>GTCGAGTTAATTTGTTAACCACCATGGATTACAAGGATCATGACATTGACTACAAA<br>GACGATGACGACAAGGGAGCCACTGGCGACGCC DNTTIP1_RT_13<br>Reverse<br>TGGTTAGCAGAGGGTTAACTCAGGTCTGTGGAGGTGCTTC DNTTIP1_RT_14
Silent mutation for<br>DNT_si1 resistance	Forward<br>CTACATCAAGCACCCACATCTCTTTAAATATGCAGCTGACCCCCAG DNTTIP1_RT_9<br>Reverse<br>CTGGGGGTCAGCTGCATATTTAAAGAGATGTGGGTGCTTGATGTAG DNTTIP1_RT_10
Silent mutation for<br>DNT_si2 resistance	Forward<br>GCTGGTTCTTACGAACCCCTGGAACATCATGATAAAGCACCGGCAG DNTTIP1_RT_11<br>Reverse<br>CTGCCGGTGCTTTATCATGATGTTCCAGGGGTTCGTAAGAACCAGC DNTTIP1_RT_12

Supplementary Table 6. Oligonucleotides used for cloning
