# Supplementary material for: The MiDAC histone deacetylase complex is essential for embryonic development and has a unique multivalent structure
Source: Nat Commun. 2020 Jun 26;11:3252. doi: 10.1038/s41467-020-17078-8 (PMC7319964; doi:10.1038/s41467-020-17078-8)
Supplement: Supplementary file 3 — Reporting Summary [file 41467_2020_17078_MOESM3_ESM.pdf]

nature research

Corresponding author(s): John Schwabe

Last updated by author(s): May 27, 2020

Reporting Summary

Nature Research wishes to improve the reproducibility of the work that we publish. This form provides structure for consistency and transparency in reporting. For further information on Nature Research policies, see Authors & Referees and the Editorial Policy Checklist .

Statistics

For all statistical analyses, confirm that the following items are present in the figure legend, table legend, main text, or Methods section.

n/a	Confirmed	
☐	☒	The exact sample size (*n*) for each experimental group/condition, given as a discrete number and unit of measurement
☐	☒	A statement on whether measurements were taken from distinct samples or whether the same sample was measured repeatedly
☐	☒	The statistical test(s) used AND whether they are one- or two-sided<br>*Only common tests should be described solely by name; describe more complex techniques in the Methods section.*
☒	☐	A description of all covariates tested
☐	☒	A description of any assumptions or corrections, such as tests of normality and adjustment for multiple comparisons
☐	☒	A full description of the statistical parameters including central tendency (e.g. means) or other basic estimates (e.g. regression coefficient) AND variation (e.g. standard deviation) or associated estimates of uncertainty (e.g. confidence intervals)
☐	☒	For null hypothesis testing, the test statistic (e.g. *F*, *t*, *r*) with confidence intervals, effect sizes, degrees of freedom and *P* value noted<br>*Give P values as exact values whenever suitable.*
☒	☐	For Bayesian analysis, information on the choice of priors and Markov chain Monte Carlo settings
☒	☐	For hierarchical and complex designs, identification of the appropriate level for tests and full reporting of outcomes
☐	☒	Estimates of effect sizes (e.g. Cohen's *d*, Pearson's *r*), indicating how they were calculated

Our web collection on statistics for biologists contains articles on many of the points above.

Software and code

Policy information about availability of computer code

Data collection	EM data was collected on a ThermoFisherTitan Krios using EPU 1.9<br>RNAseq data was collected by Novogene using an Illumina PE150, Q30; 20 million reads<br>Flow cytometry samples were analysed using a BD AccuriTM C6 flow cytometer (BD Biosciences).

Data analysis

Prism 7.0
iTASSER
PHYRE 2
Relion 3.0
Coot 0.8.9.2
CCPEM 1.3.0
Phenix 1.14
MacPymol 1.8
TRIMGALORE! v. 0.4.5 (Cutadapt v.1.11)
STAR v2.5.2b
Picard 2.6.0
Samtools v. 1.3.2
DESEQ2 v,. 1.22.2
R v. 3.4.1
Fiji (ImageJ v. 2.0.0-rc-69/1.52p)
BD Accuri C6 Plus v. 1.0.23.1
Chimera 1.14
FCSalyzer software v 0.9.15-alpha

For manuscripts utilizing custom algorithms or software that are central to the research but not yet described in published literature, software must be made available to editors/reviewers. We strongly encourage code deposition in a community repository (e.g. GitHub). See the Nature Research guidelines for submitting code & software for further information.

Data

Policy information about availability of data

All manuscripts must include a data availability statement. This statement should provide the following information, where applicable:

- Accession codes, unique identifiers, or web links for publicly available datasets
- A list of figures that have associated raw data
- A description of any restrictions on data availability

The EM maps for the dimer and tetramer are available from EMDB (EMD-11041 and EMD-11042). The coordinates for the dimer and tetramer models are available from the PDB (6Z2J and 6Z2K). The RNAseq data that support the findings of this study have been deposited in GEO with the primary accession code GSE144748. The source data underlying Figures 1, 2, and 3 and Supplementary Figures 1, 3, 4, 5, 6, 9 and 12 are provided as a Source Data file.

Field-specific reporting

Please select the one below that is the best fit for your research. If you are not sure, read the appropriate sections before making your selection.

☒ Life sciences ☐ Behavioural & social sciences ☐ Ecological, evolutionary & environmental sciences

For a reference copy of the document with all sections, see nature.com/documents/nr-reporting-summary-flat.pdf

Life sciences study design

All studies must disclose on these points even when the disclosure is negative.

Sample size

For mouse MEF embryo analysis at least 2 wildtype or homozygous embryos were used to account for technical and biological variation. For RNAseq using MEF samples at least 20000000 read pairs were generated per sample. For in vitro experiments at least 3 independent experiments were conducted to account for biological and technical variability. No statistical methods were used to determine size. Statistical tests were then performed using GraphPad to provide confidence in the conclusions made.

Data exclusions

No data was excluded from the analysis

Replication

HDAC assays, real-time PCR, quantitative wester blotting are the mean of at least 3 independent experiments
Misaligned chromosomes are from a minimum of 50 mitotic cells per independent experiment; 3 independent experiment for human cells; 3 independent for Mideas MEFs; 2 independent experiments for DNTTIP1 MEFS
RNAseq was from 3 individual Mideas MEF lines and 2 DNTTIP1 MEF lines. All attempts at replication were successful.

Randomization

Randomization was not necessary to test the effect of protein depletion on chromosome alignment, testing HDAC activity or genotyping mice as they did not need to be placed into specific groups.

Blinding

All samples were analysed identically and so investigators were not blinded during this study.

Reporting for specific materials, systems and methods

We require information from authors about some types of materials, experimental systems and methods used in many studies. Here, indicate whether each material, system or method listed is relevant to your study. If you are not sure if a list item applies to your research, read the appropriate section before selecting a response.

Materials & experimental systems

n/a	Involved in the study
☐	☒ Antibodies
☐	☒ Eukaryotic cell lines
☒	☐ Palaeontology
☐	☒ Animals and other organisms
☒	☐ Human research participants
☒	☐ Clinical data

Methods

n/a	Involved in the study
☒	☐ ChIP-seq
☐	☒ Flow cytometry
☒	☐ MRI-based neuroimaging

Antibodies

Antibodies used	Mouse anti-HDAC1 (Santa Cruz sc-81598; WB 1:400)<br>Rabbit anti-HDAC2 (Sigma 05-814; WB 1:1000)<br>Polyclonal Rabbit IgG (Abcam ab37415; IP 1ug)<br>Monoclonal Mouse IgG (Santa Cruz sc-2025; IP 1ug)<br>Goat anti-Mouse HRP (Sigma 12-349; WB 1:2000)<br>Goat anti-Rabbit HRP (Sigma 12-348; WB 1:2000)<br>Donkey anti-Goat HRP (Sigma AP180P; WB 1:5000)<br>Goat anti-Rabbit 800CW (LiCOR 925-32211; 1:10000)<br>Goat anti-Mouse 680RD (LiCOR 925-68070; 1:10000)<br>Rabbit anti-MIDEAS (Atlas HPA003111; 1ug/ml)<br>Rabbit anti-DNTTIP1 (Abcam ab174663; IF 5ug/ml; WB 2ug/ml)<br>Rabbit anti-HDAC1 (Abcam ab109411; IF 0.4ug/ml; WB 0.16ug/ml)<br>Mouse anti-FLAG (Sigma F3165; clone M2; WB 1:2000)<br>Goat anti-Lamin B (Santa Cruz (no longer available); WB 0.2ug/ml)<br>Rabbit anti-alpha Tubulin (Invitrogen PA5-85922; IF and WB 0.5ug/ml<br>Mouse anti-alpha Tubulin (Sigma F2043; clone TUB 2.1; IF 0.3ug/ml)<br>Mouse anti-CenpA (Abcam ab13939; IF 5ug/ml)<br>Goat anti-rabbit 488 (Invitrogen #A32731; IF 1:200)<br>Goat anti-mouse 594 antibodies (Invitrogen #A32742; IF 1:200)
Validation	Antibody validations are available from these websites: https://www.atlasantibodies.com/, www.abcam.com, www.sigmaaldrich.com, www.scbt.com/, www.thermofisher.com/invitrogen, https://www.licor.com/bio/. The MIDEAS antibody had previously been validated for IHC and IF. We validated for western blotting and IP using a combination of siRNA, western blotting and HDAC activity assays. The DNTTIP1 antibody had already been validated for western blotting and IP. We validated for IF using a combination of siRNA and over expressed fluorescently tagged DNTTIP1.

Eukaryotic cell lines

Policy information about cell lines

Cell line source(s)	HEK293F obtained from Invitrogen, U2OS from American Type Culture collection, Hela from American Type Culture collection
Authentication	Cell lines were not authenticated in house. We relied on the source of the original line for authenticity.
Mycoplasma contamination	Negative testing for mycoplasma
Commonly misidentified lines (See ICLAC register)	No commonly misidentified cell lines were used in the study.

Animals and other organisms

Policy information about studies involving animals; ARRIVE guidelines recommended for reporting animal research

Laboratory animals	Mice C57BL/6J, both male and female mice were used and mated from 6-26 weeks after birth

Wild animals	Study did not involve wild animals
Field-collected samples	Study did not involve field-collected samples
Ethics oversight	Experiments conformed to British Home Office Regulations under project licenses P16D64BDE and PEBDF7FCB

Note that full information on the approval of the study protocol must also be provided in the manuscript.

Flow Cytometry

Plots

Confirm that:

- [x] The axis labels state the marker and fluorochrome used (e.g. CD4-FITC).
- [x] The axis scales are clearly visible. Include numbers along axes only for bottom left plot of group (a 'group' is an analysis of identical markers).
- [x] All plots are contour plots with outliers or pseudocolor plots.
- [x] A numerical value for number of cells or percentage (with statistics) is provided.

Methodology

Sample preparation	MEFs were isolated from e13.5 embryos as described in methods. Cells were washed with PBS, fixed and permeabilised in 70% ethanol and stained with propidium iodide
Instrument	BD Accuri C6 from BD Biosciences
Software	BD Accuri C6 software used for collection, Data was analyzed using FCSalyzer software v 0.9.15
Cell population abundance	Cell sorting was not used. For cell cycle distribution cells were gated based on FS vs SS.
Gating strategy	Cells were gated based on forward and side scatter. Using a linear scale, cells stage was assigned based on well established propidium iodide fluorescence

- [x] Tick this box to confirm that a figure exemplifying the gating strategy is provided in the Supplementary Information.
